# Prevalence of positive atopy patch test in an unselected pediatric population

**DOI:** 10.1186/s12948-015-0011-2

**Published:** 2015-05-07

**Authors:** Nicola Fuiano, Giuliana Diddi, Maurizio Delvecchio, Cristoforo Incorvaia C

**Affiliations:** Pediatric Allergy Service, ASL FG Torremaggiore, I, Rome, Italy; UO “B Trambusti”, Children’s Hospital Giovanni XXIII, Bari, Italy; Allergy/Pulmonary Rehabilitation, ICP Hospital, 20100 Milan, Italy

**Keywords:** Allergy, Atopic dermatitis, Asthma, Rhinitis, Skin prick test, Atopy patch test

## Abstract

**Background:**

In the latest decades, epidemiological studies on allergic disorders in children, including atopic dermatitis, rhinitis and asthma, demonstrated a continuous increase in prevalence. However, such studies are usually performed by questionnaires and, sometimes, by skin prick test or in vitro IgE tests, while the portion of allergy sustained by the cell-mediated mechanism is neglected, because the essential test, i.e. the atopy patch test is not performed.

**Methods:**

This cross-sectional survey studied by a specific questionnaire, skin prick test and atopy patch test, an unselected population, represented by the entire scholastic population attending a Primary school and a Junior Secondary school in the rural town of San Marco in Lamis, 12.000 inhabitants (Puglia, Italy).

**Results:**

Among the 456 subjects included, 78 (17.1 %) had a positive skin prick test and 57 (12.5 %) had a positive atopy patch test. In particular, 13.4 % of subjects were positive only to skin prick test and 8.8 % were positive only to atopy patch test. The allergen most frequently positive was the house dust mite, with 41 positive results to skin prick test and 55 to atopy patch test, while for pollen positive results concerned almost exclusively the skin prick test.

**Conclusions:**

This survey on an unselected population of children detected a prevalence of positive results to atopy patch test not so distant from the positive results to skin prick test, and in 8.8 % of subjects the atopy patch test was the only positive test. This would suggest to add the atopy patch test in future epidemiological studies on allergy, in order to avoid to overlook the not negligible portion of patients with T-cell-mediated allergy.

## Introduction

The atopy patch test (APT) was introduced in the 1980s using the technique of the patch test, used for diagnosis of contact dermatitis, to test the T-cell mediated sensitization to food allergens and inhalant allergens in patients with atopic dermatitis (AD) [1]. Recent research has shown that also in patients with respiratory diseases such as rhinitis and asthma the allergic symptoms may be sustained by T-cell mediated reactions as demonstrated by positive results to APT. Importantly, in patients with T-cell-mediated allergy the APT can be the only positive test [2-5]. These clinical data on the role of the APT are supported by evidence on the capacity of such test to reproduce the pathophysiologic events of AD. In particular, the application of the APT to skin of AD patients is followed by an influx of inflammatory dendritic epidermal cells [6], being possible to detect, 24 hours after APT, a Th2 cytokine pattern, with a shift to a Th1 pattern, as occurs in chronic AD skin lesions, after 48 hours [7,8].

These capacities should suggest that the APT would be useful in epidemiologic studies on allergic diseases. Indeed, the prevalence of positive APT was evaluated only in studies on allergic subjects. In particular, a multicentric European survey on patients with AD found that APT to food and inhalant allergens was frequently positive, ranging from a maximum value of 39 % for house dust mites (HDM) to a minimum of 9 % for celery; of note, positive APT with negative skin prick test (SPT) or specific IgE in serum for the respective allergen was seen in 17 % of the patients [9]. A very recent study performed the APT in children with eosinophilic esophagitis, with positive results in around 46 % of cases [10].

Instead, epidemiologic surveys on allergy in the general population commonly use questionnaires for symptoms, SPT and specific IgE measurement [11-13], but no study employing the APT is thus far available. We evaluated the prevalence of positive APT and SPT in an unselected population of subjects in pediatric age.

## Methods

### Patients

This cross-sectional study included the entire scholastic population attending a Primary school and a Junior Secondary school in a rural town in Southern Italy (San Marco in Lamis, 12.000 inhabitants, Puglia). During the 2009–2010 school year we recruited 459 pupils and students [246 males (53.6 %), 213 females (46.4 %), age range 7.3-13.9 years, mean age 10.8 ± 1.5 years; median age 11.0 years]

The study was approved by written informed consent from the Headmaster of the School and also from all the recruited subject’s parents.

### Methods

We gave each and every one of the pupil’s parents a written questionnaire (WQ), consisting of 4 pages. This questionnaire had been introduced by Peroni et al. and used in previous epidemiological studies [12]. The WQ contained the three core ISAAC (International Study on Asthma and Allergy in Children) modules asking about the diagnosis of atopic dermatitis, allergic rhinitis, wheezing, and asthma [11] according to a precise diagnosis performed by a pediatrician or general practitioner or hospital doctor. In particular, the items of the questionnaire addressed the presence (either long-lasting or recent, i.e. in the last 12 months) of signs and symptoms of AD (eczema), rhinitis and asthma.

The questionnaire was delivered directly and personally to the parents to be filled in at home and returned when completed in a sealed envelope. After written and signed consent by the parents had been received, we submitted all the study subjects to SPT and APT using the most common aeroallergens in our geographic area: pollens (grasses, *Parietaria*, cypress, Compositae), house dust mites, cat epithelium, and *Alternaria tenuis*.

For SPT we used diagnostic material from Stallergenes (Antony, France), testing as negative control glicerosalin solution and as positive control histamine (10 mg/ml), according to the guidelines of the European Academy of Allergy and Clinical Immunology (EAACI) [14]; the reactions were considered positive in the presence of a wheal diameter of at least 3 mm and larger than the negative control.

For APT, we used the diagnostic material from Chemotecnique (Wellinge, Sweden). The substance to be tested was applied onto intact skin of the lower back and held firmly in position using an adhesive patch test made up of an aluminum Finn chamber 8 mm in diameter, with an area of 50 mm^2^ and a volume of about 20 μl.

The application period was 48 h. The test was read no less than 30 min after removal to avoid margin effect. Results were interpreted according to the American Academy of Dermatology for APT, with a scale ranging from 1+ (weak reaction) to 3+ (strong reaction) [15]. Only reactions of 2+ and 3+ were considered positive for the purpose of the study. Table 1 shows the list and position of the allergens tested. The Finn Chamber with Petrolatum was used as the control test. We made sure that the families had been informed that they should avoid giving antihistamines to the children during the three-day period preceding the application of APT. We also advised them not to apply creams/ointments containing corticosteroids in the week before the test.

**Table 1:** **Allergens used for APT**

**Position**	**Allergen**	**gr ± 10 %**
1	Alternaria	0,085
2	Cat epithelium	0,086
3	Grass pollen	0,084
4	House dust mites (Dermatophagoides)	0,085
5	Compositae pollen	0,086
6	Cypress pollen	0,088
7	Parietaria pollen	0,088
8	Petrolatum control	0,085

### Statistical analysis

Data are presented as percentages and as mean ± SD for qualitative and quantitative variables, respectively. Statistical analysis was performed using *χ*^2^ test for categorical variables. Contingency tables were used to analyze the association between the categorical variables and the tests used in the study (APT and SPT). Statistical significance was assumed for p < 0.05. The positive and negative predictive values were calculated as appropriate and the limits of the 95 % confidential interval displayed (CI95%).

## Results

Of the 459 questionnaires delivered to the families, 456 (244 males, 212 females) were returned. Table 2 shows the clinical pictures according to age and gender obtained from the answers to the questionnaire.

**Table 2:** **Distribution of the studied population according to the different clinical expressions of allergy as assessed by questionnaires**

	**Atopic dermatitis**		**Rhinitis**		**Asthma/wheezing**	
subjects N°	66		109		66	
males:females	43:23		66:43		36:30	
age range (years)	7.8 – 13.2		7.8 – 13.2		8.2 – 13.3	
mean age ± DS	10.9 ± 1.5		10.7 ± 1.6		11.3 ± 1.3	
	**Ever**	**Last 12 months**	**Ever**	**Last 12 months**	**Ever**	**Last 12 months**
subjects N° (%)	65	1	104	5	66	0
males: females	42:23	1:0	63:41	3:2	36:30	---
age range (years)	7.8 – 13.2	---	7.8 – 13.2	12.2 – 13.2	8.2 – 13.3	---
mean age ± DS	10.9 ± 1.5	---	10.7 ± 1.6	12.5 ± 0.4	11.3 ± 1.3	---

Of the 456 questionnaires returned, 279 (61.2 %) were negative for any sign or symptom, while 177 (38.8 %) were positive for symptoms of AD, rhinitis, or asthma/wheezing. In particular, 129 (28.3 %) were positive for only one kind of symptoms and 48 (10.5 %) were positive for more than one kind of symptoms. Overall, there were 66 questionnaires positive for AD, 109 for rhinitis, and 66 for asthma/wheezing. Most signs and symptoms were long-lasting, while only in 6 subjects the onset of signs and symptoms was in the last 12 months. In the 279 subjects with negative history, 29 (10.4 %) had a positive SPT and 26 (9.3 %) had a positive APT. In the 177 subjects with positive history, 49 (27.7 %) had a positive SPT and 31 (17.5 %) had a positive APT.

In the overall study population of 456 subjects, 78 (17.1 %) had a positive SPT and 57 (12.5 %) had a positive APT. In particular, 61 subjects (13.4 %) were positive only to SPT and 40 (8.8 %) were positive only to APT. The rate of positive test was significantly higher in males than in females both for SPT (22.1 % vs. 11.3 %, p = 0.002) and APT (15.6 % vs. 9 %, p = 0.033). No positive result to control tests occurred, both for glicerosalin solution in SPT and for petrolatum control for APT.

**Figure 1:**
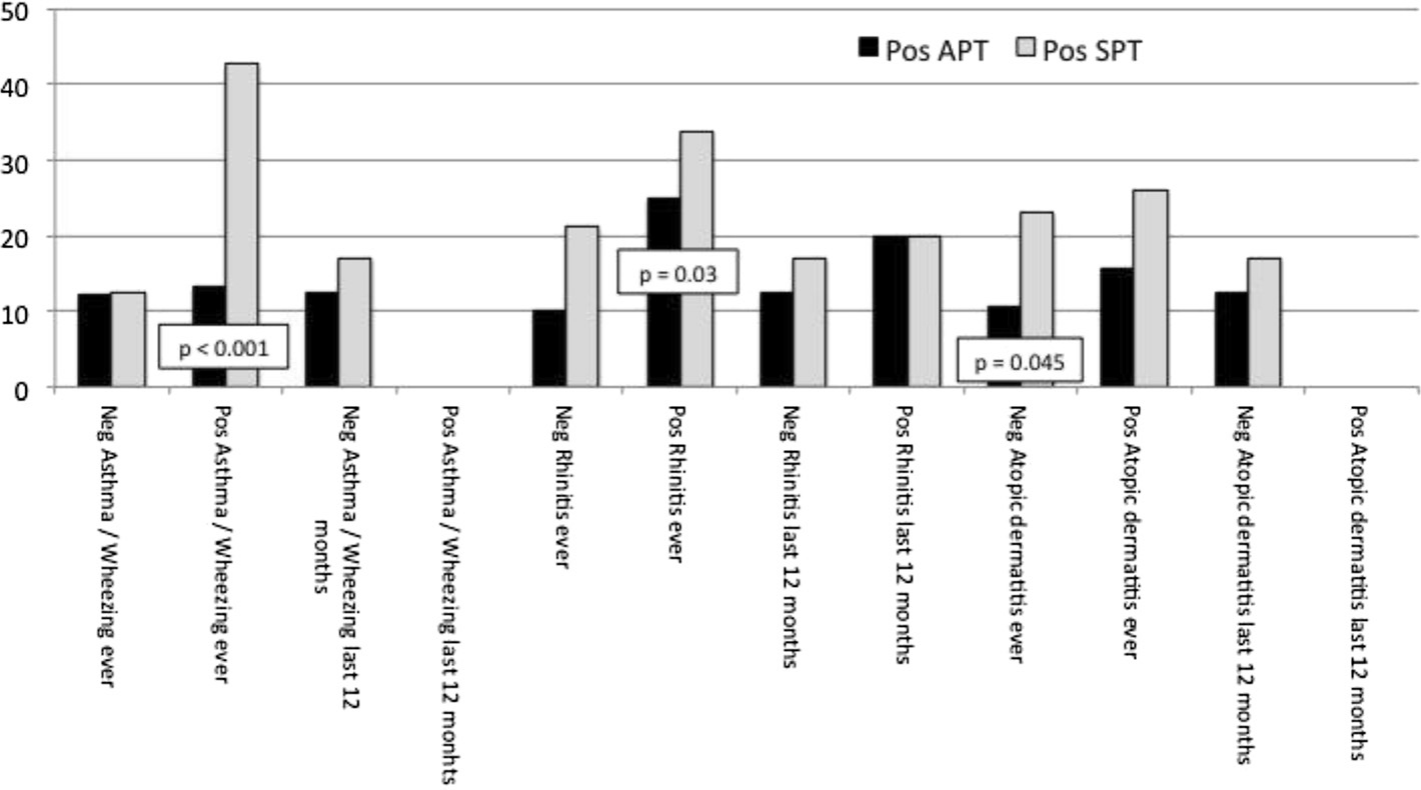
**Rate of positive APT and positive SPT according to the history data.**

Figure 1 shows the results to APT and SPT according to the different clinical history. Significant differences were found for a higher rate of positive SPT concerning subjects with a history of asthma/wheezing ever, with a history of rhinitis ever, and with a negative history of AD ever. Table 3 shows the results to the single allergens tested. The allergen most frequently positive was the house dust mite, with 41 positive results to SPT and 55 to APT.

**Table 3:** **Results to APT and SPT for the single allergens tested**

	**Positive SPT**	**Positive APT**
Alternaria tenuis	8	0
Cypress pollen	29	0
Compositae pollen	4	2
Dermatophagoides	41	55
Cat epithelium	9	0
Grass pollen	28	3
Parietaria pollen	4	1

## Discussion

Epidemiology is a powerful tool in investigating the importance of allergic diseases. Studies conducted lately have shown a continuous increase in the prevalence of allergy. In particular, the important ISAAC study focused on three major allergic diseases (asthma, rhinitis, and AD) and included a large number of countries worldwide. It detected in the Phase Three [16], which was completed a mean of 7 years after Phase One, an increased prevalence for at least one disorder. Increases were twice as common as decreases, and were more common in the 6–7 year age-group than in the 13–14 year age-group. For both age-groups, the majority of participating centres showed increases in all three disorders. All epidemiological surveys on allergy are based on questionnaires addressing the common symptoms of allergy and, quite often, on diagnostic tests such as SPT or specific IgE measurement. However, it is known since the classification by Coombs and Gell of the immunopathologic mechanisms [17] that the IgE-mediated mechanism drives a great part of allergies, but also other mechanisms are important. This was finally acknowledged in the Report of the Nomenclature Review Committee of the World Allergy Organization by Johansson et al. [18]. Among the different mechanisms, the T-cell-mediated one has long been recognized as decisive in AD, this attributing to the APT a significant diagnostic role, initially shown for sensitization to foods [19] but later also for sensitization to inhalant allergens and particularly to house dust mites [20,21]. The recent research further expanded the clinical importance of APT, based on the demonstration that this test is positive also in patients with respiratory allergy [3-5,22]*.* The fact that the APT may be the only positive test in patients with respiratory allergy underlines the importance of including this test in the diagnostic work-up of allergy. Otherwise, patients with negative results to SPT or in vitro IgE tests may be erroneously classified as nonallergic. Adding the APT allows us to achieve a correct diagnosis [23]. As far as the use of diagnostic tests in epidemiologic surveys is concerned, no studies using the APT are available other than those performed in populations of patients already known as allergic [9,10]. The present study shows that a positive APT is quite common in an unselected pediatric population, formed by all subjects attending Primary School and Junior Secondary School in a small Southern Italian town. Among the 456 subjects included in the survey, 17.1 % had a positive SPT and 12.5 % had a positive APT. The prevalence of positive SPT must be compared with epidemiological studies in the latest decade. In the European Community Respiratory Health Survey on adult subjects there was a wide variation in the prevalence of sensitization in the different countries, with a median between centres of 21.7 % for dust mite, 16.9 % for grass pollen, and 8.8 % for cat [24]. The recent study by Blomme et al. found in an unselected population in Ghent (Belgium), of age ranging from 3 to 86 years, an overall prevalence of positive SPT to common aeroallergens of 40.3 %, with a clinical diagnosis of allergic rhinitis in 30.9 % of cases. The peaks of prevalence were observed in the third and fourth decades of life, but also in pediatric age the prevalence of positive SPT was higher than 25 % [25]. The 17.1 % positive SPT rate we observed is lower, but the environment where our survey was performed may explain this difference. San Marco in Lamis is a small town of 12,000 inhabitants in a rural area, while Ghent, with around 245,000 inhabitants is Belgium's second largest municipality. A rural environment, like that of San Marco in Lamis, was found to be less favorable than urban areas for developing allergic diseases [26]. Analyzing only the subjects with symptoms, the rate of positive SPT was around 28 %. This value is comparable with data from previous studies on unselected populations of children [27,28], but is obviously much lower than observed in patients referring to specialists for allergological evaluation.

The findings on positive APT (9.3 %) in subjects with negative history and 17.5 % in subjects with positive history cannot be compared with previous epidemiological data, because there are no studies that limited the investigation to APT results to only inhalant allergens. However, we can confirm the observations from clinical studies [5,9,21] that APT may give positive results in concordance with SPT but may also be the only positive test (3.7 % and 8.8 % in the present study, respectively). The major role played by the house dust mite was also confirmed, with positive results accounting for 33.3 % of the overall positivity to SPT and to 91.1 % of the overall positivity to APT. Instead, in our study sensitization to pollens was almost exclusively IgE-mediated.

## Conclusion

In this survey on an unselected population of children we observed a prevalence of positive results to APT not so distant from the positive results to SPT, especially concerning dust mites, and in 8.8 % of subjects the APT was the only positive test. This would suggest that by adding the APT in future epidemiological studies on allergy, would mean avoiding the not negligible portion of patients with T-cell-mediated allergy being overlooked.

## Abbreviations

APT: Atopy patch test
AD: Atopic dermatitis
HDM: House dust mites
SPT: Skin prick test
WQ: Written questionnaire
ISAAC: International Study on Asthma and Allergy in Children
EAACI: European Academy of Allergy and Clinical Immunology
