# Erratum: Prevalence of positive atopy patch test in an unselected pediatric population

**DOI:** 10.1186/s12948-015-0024-x

**Published:** 2015-07-07

**Authors:** Nicola Fuiano, Giuliana Diddi, Maurizio Delvecchio, Cristoforo Incorvaia

**Affiliations:** Pediatric Allergy Service, ASL FG Torremaggiore, I, Rome, Italy; UO “B Trambusti”, Children’s Hospital Giovanni XXIII, Bari, Italy; Allergy/Pulmonary Rehabilitation, ICP Hospital, Milan, 20100 Italy

## Erratum

Following publication of our article [1] we have noticed that the corresponding author name ‘Cristoforo Incorvaia’ was incorrectly listed and contained an erroneous C after the author’s last name. The author list has now been corrected.

## References

[1] Fuiano N, Diddi G, Delvecchio M, Incorvaia C (2015). Prevalence of positive atopy patch test in an unselected pediatric population. Clin Mol Allergy.

